# Pharmacokinetics of Subcutaneous Dupilumab Injection With an Autoinjector Device or Prefilled Syringe

**DOI:** 10.1002/cpdd.1073

**Published:** 2022-03-12

**Authors:** Yehuda Z. Cohen, Xiaojia Zhang, Binfeng Xia, Matthew P. Kosloski, Mohamed A. Kamal, John D. Davis, Vanaja Kanamaluru, Christine Xu

**Affiliations:** ^1^ Sanofi Bridgewater New Jersey USA; ^2^ Sanofi Beijing China; ^3^ Regeneron Pharmaceuticals, Inc. Tarrytown New York USA; ^4^ Present address: Boehringer Ingelheim, Tokyo, Japan.

**Keywords:** bioequivalence, dupilumab, subcutaneous injection device

## Abstract

Dupilumab, a human monoclonal antibody against interleukin‐4 receptor alpha, has demonstrated efficacy and an acceptable safety profile in adult and pediatric patients with moderate‐to‐severe atopic dermatitis (AD) and other type 2 inflammatory diseases. Dupilumab is available in 200‐ and 300‐mg strengths as a prefilled syringe with a needle shield (PFS‐S), and more recently as an autoinjector (AI) device. This study was designed to assess the pharmacokinetic (PK) comparability of a single subcutaneous (SC) dose of dupilumab 200 mg, delivered by 2 different devices, AI (test) versus PFS‐S (reference). A total of 130 healthy male and female participants were enrolled in this phase 1 parallel design study, with 128 evaluable for PK. Following dupilumab 200‐mg SC injection, dupilumab exposure in serum was similar for both AI and PFS‐S. The geometric mean ratios of PK parameters with 90% confidence intervals were 1.08 (0.97‐1.21) for maximum serum concentration (C_max_) and 1.11 (0.96‐1.28) for area under the serum concentration–time curve until the last quantifiable concentration (AUC_last_). Dupilumab administration by both devices was well tolerated, and there were no serious adverse events, or severe treatment‐emergent adverse events experienced during the study. Overall, exposure to dupilumab 200 mg was comparable when administered via the AI or PFS‐S devices in healthy male and female study participants.

Atopic dermatitis (AD), a chronic and relapsing, type 2 inflammatory disease, involves skin lesions and pruritus that can significantly impair quality of life.^1^ AD is estimated to affect 20% of children and 2% to 8% of adults worldwide.^2^, ^3^ Dupilumab is a fully human monoclonal antibody that blocks interleukin (IL)‐4 receptor alpha, the shared receptor component for IL‐4 and IL‐13, inhibiting signaling of both IL‐4 and IL‐13, which are key and central drivers of type 2–mediated inflammation in multiple diseases.^4^, ^5^ In phase 3 randomized trials, dupilumab has demonstrated efficacy and an acceptable safety profile in adult and pediatric patients with moderate‐to‐severe AD and other type 2 inflammatory diseases such as asthma and nasal polyps.^5^, ^6^, ^7^, ^8^, ^9^, ^10^, ^11^, ^12^, ^13^, ^14^, ^15^

Dupilumab exhibits nonlinear target‐mediated pharmacokinetics (PK).^16^ It is well absorbed after subcutaneous (SC) administration (bioavailability of 61%; time to reach maximum serum concentration [C_max_] of 3‐7 days after a single dose), exhibits a low volume of distribution (4.12 L) indicating distribution largely in the vascular compartment, and undergoes elimination by parallel linear and nonlinear pathways.^16^, ^17^ At higher systemic concentrations, elimination is predominantly through the linear, nonsaturable proteolytic pathway, while at lower concentrations the nonlinear saturable target‐mediated elimination pathway predominates. Body weight is the primary responsible variable in dupilumab PK, with lower exposure observed at higher body weight.^16^, ^17^, ^18^

Dupilumab is currently available for SC administration as a prefilled syringe with a needle shield (PFS‐S) and an autoinjector (AI) device in 200‐ and 300‐mg dose strengths. Drug delivery by the AI (also known as a prefilled pen) has been shown, for other drugs, to be preferred by some patients, and can be administered by patients or caregivers, and thus may increase compliance over long treatment durations. Different administration devices may affect bioavailability; therefore, this study was designed to assess the PK comparability of dupilumab exposure when administered via the AI versus PFS‐S devices.

Suitable methods used to assess PK comparability include studies as part of a patient clinical trial or a stand‐alone PK study in healthy participants. The latter option was chosen for this study as this allows for a more controlled setting where potential sources of PK variability such as population, injection site, weight, and self‐administration technique can be reduced. Accordingly, the study was conducted in healthy participants within a prespecified body weight range, with SC administration of dupilumab performed by trained clinical staff via a single site of injection (abdomen). Thus, the study was designed to determine if the AI device, when used as intended, delivers a dose of dupilumab that results in comparable dupilumab exposure as would be obtained when using the PFS‐S, according to current regulatory guidance for conducting device‐bridging studies.

## Methods

### Study Design

This study was a phase 1, single‐center study conducted in an open‐label, randomized, parallel design. The study was conducted between November 6, 2019, and January 6, 2020, at 1 study site, Clinical Pharmacology of Miami, Miami, Florida. An independent institutional review board (Integ Review, Austin, Texas) approved the protocol, and the study was conducted in accordance with the Declaration of Helsinki and the International Council for Harmonisation guidelines for Good Clinical Practice. All participants provided written informed consent before the study.

A parallel design was chosen due to the low clearance of dupilumab (typical of monoclonal antibodies after the target‐mediated pathway is saturated), which leads to prolonged exposure in serum, and also due to the known possibility of the development of antidrug antibodies, making a crossover design impractical. The study was conducted in healthy individuals, as they demonstrate similar dupilumab PK as patients with AD and other type 2 inflammatory diseases.^16^, ^17^ The primary objective of the study was to compare the systemic exposure of dupilumab after a single dose of dupilumab 200 mg SC when administered by AI relative to PFS‐S. The secondary objective was to determine the safety and tolerability of a single injection of dupilumab 200 mg when administered by the 2 different devices.

Planned enrollment for the study was 130 participants. The sample size calculation was estimated based on a standard deviation (SD) of up to 0.55 of PK exposure parameters. With 120 total subjects (60 subjects per treatment group) and a true total SD of 0.55, the PK parameters adjusted geometric mean ratios between treatments were expected to be estimated with a maximum imprecision of 16.5% (ie, the 90%CI would be no wider than 0.835 to 1/0.835 times the observed ratio) with 90% assurance. Thus, if the observed ratio was determined to be 1.05, the 90%CI was to be no wider than 1.05*0.835 (ie, 0.88) to 1.05/0.835 (ie, 1.26). Approximately 130 subjects were randomized to achieve at least 120 subjects with PK sample collection through day 43. Key inclusion criteria were aged 18 to 65 years, body weight of 70 to 100 kg, and certification as healthy following clinical assessment. Key exclusion criteria included presence of relevant medical conditions (as assessed by the investigator), headaches, recent blood donation (within 2 months), prior drug or biologic sensitivity, alcohol or drug abuse, pregnancy, asthma, recent (within 4 months) use of biologics, or any prior exposure to dupilumab.

Each participant received a single dose of dupilumab 200 mg SC (volume delivered was 1.14 mL of a 175 mg/mL solution) administered in the abdomen by either the PFS‐S or AI device by study site staff. PFS‐S was administered as a skin pinch using a 45° angle, the AI device was administered at a 90° angle to skin without a pinch. The rationale for using trained staff to administer the drug to a single injection site was to ensure that the administration was performed in a controlled, standardized manner. Blood samples for PK were taken from the participants before injection (baseline to day 1) and on days 2, 4, 8, 11, 15, 22, 29, 36, and 43.

### Pharmacokinetics and Statistical Analysis

All participants with evaluable PK data were included in the PK population. The C_max_ and time to reach C_max_ of dupilumab were obtained directly from experimental observations. Area under the serum concentration–time curve until the last quantifiable concentration (AUC_last_) was calculated by a noncompartmental approach using the linear trapezoidal method. PK parameters were summarized (mean, geometric mean, median, standard deviation, minimum, and maximum) and listed for each treatment group (200 mg AI [test] or 200 mg PFS‐S [reference]).

For C_max_ and AUC_last_, point estimates of the geometric mean ratio, along with 90%CIs, for the 200 mg AI relative to the 200 mg PFS‐S were obtained with a linear fixed effects model.

### Analytical Methods

Serum samples for quantitation of functional dupilumab were analyzed using a validated enzyme‐linked immunosorbent assay (ELISA). In this functional assay, dupilumab was used as the assay standard and human IL‐4 receptor alpha served as the capture reagent.

Concentrations of dupilumab with either 1 or 2 unoccupied binding sites were measured (functional drug). The lower limit of quantitation (LLOQ) of functional dupilumab is 0.078 mg/L in undiluted human serum (Regeneron Pharmaceuticals, Inc., Tarrytown, New York).^17^, ^19^

### Safety

All participants who were exposed to at least 1 dose of dupilumab, regardless of the amount of treatment administered, were included in the safety population. The safety analysis included treatment‐emergent adverse events (TEAEs), laboratory parameters, and vital signs and was based on individual values and descriptive statistics.

## Results

### Participant Demographics and Baseline Characteristics

A total of 130 participants were enrolled in the study, 128 had evaluable PK data, and 125 completed all study visits. Summary statistics of baseline characteristics are presented in Table 1: 65 participants were enrolled into the PFS‐S treatment group and 65 into the AI treatment group.

**Table 1:** Demographics and Participant Characteristics at Baseline for the Safety Population

Characteristic	Dupilumab 200 mg AI (n = 65)	Dupilumab 200 mg PFS‐S (n = 65)	All Participants (n = 130)
Age, y, mean (SD)	46.0 (11.9)	46.7 (12.7)	46.4 (12.3)
Sex, male, n (%)	22 (33.8)	21 (32.3)	43 (33.1)
Race, n (%)			
Black or African American	14 (21.5)	9 (13.8)	23 (17.7)
White	51 (78.5)	56 (86.2)	107 (82.3)
Weight (kg), mean (SD)	80.96 (8.74)	80.28 (8.36)	80.62 (8.53)
BMI (kg/m^2^), mean (SD)	29.67 (3.12)	29.74 (3.50)	29.71 (3.30)

AI, autoinjector; BMI, body mass index; PFS‐S, prefilled syringe with a needle shield; SC, subcutaneous; SD, standard deviation.

Forty‐three participants (33.1%) were men, and 87 (66.9%) were women. All were between the ages of 19 and 65 years, and the mean age of participants included was 46.4 years. Participants were predominantly White (82.3%) and had a mean body mass index (BMI) of 29.71. Overall, the demographics and baseline characteristics were balanced between groups (Table 1).

### Pharmacokinetic Analysis

Two participants were excluded from the PK population: 1 participant in the PFS‐S treatment group had only 3 PK samples, which was insufficient for PK analysis, and 1 participant in the AI treatment group was reported not to have received the complete injection volume, as leakage from the device was noted after the device was lifted from the skin. A subsequent investigation showed no deficiencies with the device. AUC_last_ was not reported in 3 additional participants (2 in the AI treatment group, 1 in the PFS‐S treatment group) due to missing PK samples; these participants were included in evaluation of C_max_.

**Figure 1:**
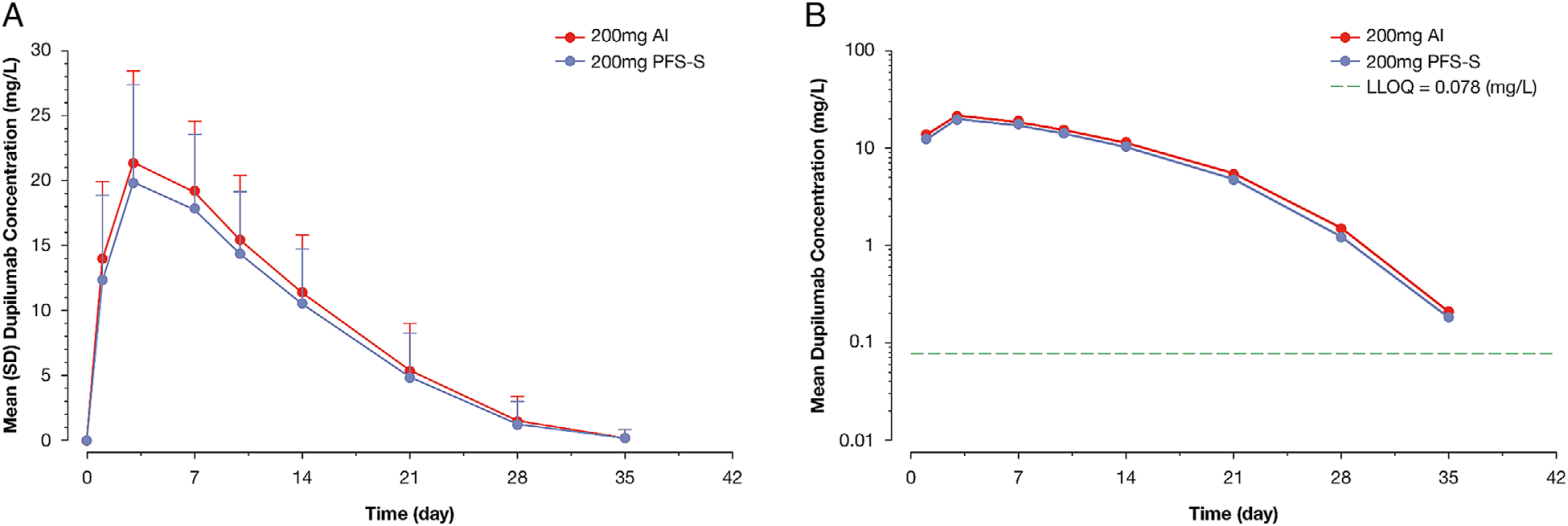
Mean dupilumab concentration–time profile following a single 200‐mg SC dose via the AI and the PFS‐S. (A) Linear plot with SD and (B) semilog plot. All BLQ values were set to 0 for the purpose of calculation of means. AI, autoinjector; BLQ, below limit of quantification; LLOQ, lower limit of quantification; PFS‐S, prefilled syringe with a needle shield; SC, subcutaneous; SD, standard deviation.

**Figure 2:**
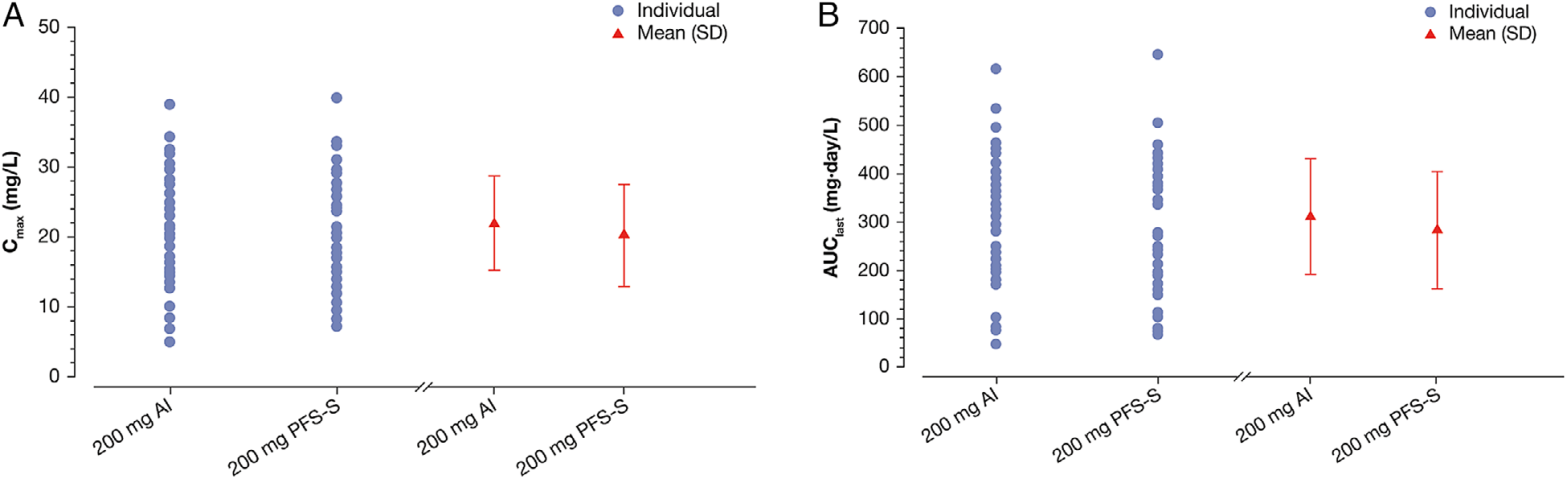
(A) Individual and mean (SD) dupilumab C_max_ values following a single 200‐mg SC dose via the AI and the PFS‐S and (B) individual and mean (SD) dupilumab AUC_last_ values following a single 200‐mg SC dose via the AI and the PFS‐S. AI, autoinjector; AUC_last_, area under the serum concentration–time curve until the last quantifiable concentration; C_max_, maximum serum concentration; PFS‐S, prefilled syringe with a needle shield; SC, subcutaneous; SD, standard deviation.

Descriptive statistics for dupilumab PK parameters in serum are provided in Table 2. After administration of dupilumab 200 mg SC, dupilumab exposure was similar for AI and PFS‐S (Figure 1). The concentration‐time profile of dupilumab was characterized by a short absorption/distribution phase, a linear β phase (indicating saturation of the target‐mediated pathway), and a terminal nonlinear target‐mediated elimination phase, which is consistent with the elimination of dupilumab by parallel linear and nonlinear pathways. Hence, values for half‐life and area under the serum concentration–time curve (AUC_last_) extrapolated to infinity were not calculated. Individual and mean C_max_ and AUC_last_ of dupilumab are presented for each treatment in Figure 2A and B, respectively. The geometric mean treatment ratios for AI and PFS‐S were 1.08 (90%CI, 0.97‐1.21) for C_max_ and 1.11 (90%CI, 0.96‐1.28) for AUC_last_ (Table 2). A sensitivity analysis demonstrated similar geometric mean ratios when the single participant reported to have received an incomplete AI injection was included (not shown).

**Table 2:** Overview of PK Parameters of Dupilumab Following a Single SC Dose via the AI and the PFS‐S

	Parameter (Units)		
	C_max_ (mg/L) Mean ± SD (Geometric Mean)	t_max_ (Day) Median (Min‐Max)	AUC_last_ (mg • day/L) Mean ± SD (Geometric Mean)
Dupilumab 200 mg AI [test] (n = 64)	21.7 ± 6.83 (20.5)	3.00 (2.96–7.02)	311 ± 118^a^ (284)
Dupilumab200 mg PFS‐S [reference] (n = 64)	20.3 ± 7.22 (18.9)	3.00 (2.92‐7.06)	284 ± 121^b^ (256)
Geometric mean (ANOVA) ratio 200 mg AI versus 200 mg PFS‐S Point estimate (90%CI)	1.08 (0.97–1.21)		1.11 (0.96–1.28)

AI, autoinjector; ANOVA, analysis of variance; AUC_last_, area under the serum concentration–time curve until the last quantifiable concentration; C_max_, maximum serum concentration; PFS‐S, prefilled syringe with a needle shield; PK, pharmacokinetics; SC, subcutaneous; SD, standard deviation; t_max_, time to maximum serum concentration.

^a^ n = 62 AUC_last_ was not reported for 2 participants due to early discontinuation (missing samples at the last 3‐4 time points).

^b^ n = 63 AUC_last_ was not reported for 1 participant due to missing samples on days 11 and 15.

### Safety Assessment

All 130 participants were included in the safety population. Overall, 5 participants experienced a total of 5 TEAEs during the study (Table 3). These included 1 occurrence of hordeolum and pregnancy in the PFS‐S treatment group, and diarrhea, dermatitis, and dysmenorrhea in the AI treatment group. The pregnancy was discovered at the end‐of‐study visit (the date of the participant's last menses was on day 18) and was ongoing at the end of the study. There were no severe TEAEs, serious adverse events [SAEs], or deaths. There were no clinically relevant abnormalities in clinical laboratory parameters or vital sign parameters during the study. Furthermore, no participants required concomitant medications during the study.

**Table 3:** Overview of TEAEs in the Safety Population Following a Single SC Dose via the PFS‐S and the AI

	Dupilumab 200 mg PFS‐S (n = 65)	Dupilumab 200 mg AI (n = 65)
Any TEAE, n (%)	2 (3.1)	3 (4.6)
Severe TEAE, n (%)	0	0
TEAE of special interest, n (%)	1 (1.5)^a^	0
Eyelid infection, n (%)	1 (1.5)	0
Dermatitis, n (%)	0	1 (1.5)
Pregnancy, n (%)	1 (1.5)	0
Dysmenorrhea, n (%)	0	1 (1.5)
Diarrhea, n (%)	0	1 (1.5)

AI, autoinjector; PFS‐S, prefilled syringe with a needle shield; SC, subcutaneous; TEAE, treatment‐emergent adverse event.

^a^ The TEAE of special interest was pregnancy.

## Discussion

In this study, we performed a controlled, stand‐alone PK study in 130 healthy participants to demonstrate PK comparability of dupilumab 200 mg SC administered using 2 different devices, a PFS‐S or the more recently introduced AI device. Both the AI and PFS‐S use the same bulk‐prefilled syringe with the same formulation and same amount of drug. The 2 presentations include the same needle length and needle diameter and are designed to target the same needle injection depth to deliver an SC injection with the same volume for each 200‐mg dose.

SC administration of biologics shows a wide range of bioavailability from 24% to 100%.^20^ After SC administration, absorption involves convection and diffusion of biologics from interstitial tissues to the lymphatic capillaries and surrounding blood vessels.^20^, ^21^, ^22^ Changes in manufacturing process, formulation, or device presentation may alter SC absorption, depending on the extent of difference(s) between the pre‐ and postchange products.^20^, ^22^ Administration with AI and PFS devices may differ with respect to orientation of the needle upon injection and injection depth, which could impact the SC absorption process.^20^ PK comparability studies conducted to compare systemic exposure of different drug products or device presentations are important to ensure that patients receive a consistent exposure to the drug.^20^

Crossover study designs, in which administration of both test and reference products to each study participant with an interceding washout period, control the contribution of intersubject variability on the comparison and are traditionally used for small‐molecule drugs with short half‐lives. Due to the long circulation time of monoclonal antibodies and potential to develop an antidrug antibody response that may impact PK between test and reference product administrations, parallel study designs in which each patient receives either the test or reference product, but not both, are favored over a crossover design.

A recent review to understand the current practices in bridging SC device presentations identified 17 biologics approved by the FDA's Center for Drug Evaluation and Research with PK comparability assessments for PFS and AI presentations.^20^ Although not all PK comparability studies in the review were powered for demonstrating bioequivalence (BE), most studies conducted for approved AI presentations met traditional BE criteria (90%CIs of the geometric mean ratios for key PK parameters of the test product relative to the reference to fall between 0.80 and 1.25). Injection depth of AI presentation and the injection site of the AI or the PFS were found to be potential factors affecting drug exposure and hence the comparability outcome. Multiple parameters in PK study design can be leveraged to minimize PK variability not due to the devices, such as using a single injection site in all participants, which can enhance the power of demonstrating comparable PK.^20^ Additionally, a study in healthy subjects is more sensitive in evaluating similarity because it is likely to produce less PK variability compared with a study in patients with potential confounding factors such as underlying and/or concomitant disease, concomitant medications, and self‐administration technique. Accordingly, the current dupilumab study was conducted in a controlled setting in healthy participants. In this setting, dupilumab was administered by the well‐trained clinical site staff to reduce potential self‐medication dosing errors, and a single site and body location for injection was used to minimize variability. Since body weight is an influential covariate for dupilumab PK, its impact in the context of a parallel study design was also minimized by enrolling participants in a limited weight range (70‐100 kg).^16^, ^17^

The study found that systemic dupilumab exposure after a single dose of dupilumab 200 mg SC administered by AI was similar to that of the PFS‐S in healthy male and female participants. The point estimate of C_max_ and AUC_last_ and 90%CI of C_max_ were within the range of 0.80 to 1.25, while the upper bound of the 90%CI of AUC_last_ (1.28) was slightly higher than 1.25. The number of subjects in this study was selected to provide a robust comparison of dupilumab devices, but the study was not specifically powered to meet BE criteria for all PK end points. Given the wide therapeutic window of dupilumab and lack of exposure‐related adverse events, the small differences in the distribution of AUC_last_ are not considered clinically relevant. These data indicate that the PK of the 2 devices was largely similar.

Dupilumab is generally well tolerated and is currently approved for patients with type 2 inflammatory diseases, including AD, asthma, and chronic rhinosinusitis with nasal polyps. Unlike other biologics that target immune pathways, dupilumab has not been shown to increase the risk of infection and reduced the risk of serious or severe infections in patients with AD.^16^, ^23^, ^24^ Previous phase 1 single‐dose dupilumab studies in healthy participants showed dupilumab was well tolerated, with an acceptable safety profile at doses up to 600 mg SC or 12 mg/kg intravenous, with the PK parameters obtained consistent with those found in the present study.^16^ Dupilumab administration by the SC route via both devices was well tolerated in this study, and there were no serious adverse events or severe TEAEs reported.

This study confirmed the comparable bioavailability and safety of dupilumab when administered with either a PFS‐S or AI device.

## Conclusions

Overall, administration of dupilumab 200 mg SC via AI resulted in exposure comparable to administration via PFS‐S in healthy male and female participants. There were no differences in the safety profile of dupilumab with either administration device in this study.

## Conflicts of Interest

Y.Z.C., B.X., V.K., and C.X. are employees and may hold stock and/or stock options in Sanofi. X.Z. is a former employee and may hold stock and/or stock options in Sanofi. M.P.K., M.A.K., and J.D.D. are employees and shareholders of Regeneron Pharmaceuticals, Inc.

## Funding

This study was funded by Sanofi and Regeneron Pharmaceuticals, Inc.
